# Phenotypic Variation and Fitness in a Metapopulation of Tubeworms (*Ridgeia piscesae* Jones) at Hydrothermal Vents

**DOI:** 10.1371/journal.pone.0110578

**Published:** 2014-10-22

**Authors:** Verena Tunnicliffe, Candice St. Germain, Ana Hilário

**Affiliations:** 1 Department of Biology, University of Victoria, Victoria, British Columbia, Canada; 2 School of Earth & Ocean Sciences, University of Victoria, Victoria, British Columbia, Canada; 3 Departamento de Biologia and Centro de Estudos do Ambiente e do Mar, Universidade de Aveiro, Campus de Santiago, Aveiro, Portugal; Universität Göttingen, Germany

## Abstract

We examine the nature of variation in a hot vent tubeworm, *Ridgeia piscesae*, to determine how phenotypes are maintained and how reproductive potential is dictated by habitat. This foundation species at northeast Pacific hydrothermal sites occupies a wide habitat range in a highly heterogeneous environment. Where fluids supply high levels of dissolved sulphide for symbionts, the worm grows rapidly in a “short-fat” phenotype characterized by lush gill plumes; when plumes are healthy, sperm package capture is higher. This form can mature within months and has a high fecundity with continuous gamete output and a lifespan of about three years in unstable conditions. Other phenotypes occupy low fluid flux habitats that are more stable and individuals grow very slowly; however, they have low reproductive readiness that is hampered further by small, predator cropped branchiae, thus reducing fertilization and metabolite uptake. Although only the largest worms were measured, only 17% of low flux worms were reproductively competent compared to 91% of high flux worms. A model of reproductive readiness illustrates that tube diameter is a good predictor of reproductive output and that few low flux worms reached critical reproductive size. We postulate that most of the propagules for the vent fields originate from the larger tubeworms that live in small, unstable habitat patches. The large expanses of worms in more stable low flux habitat sustain a small, but long-term, reproductive output. Phenotypic variation is an adaptation that fosters both morphological and physiological responses to differences in chemical milieu and predator pressure. This foundation species forms a metapopulation with variable growth characteristics in a heterogeneous environment where a strategy of phenotypic variation bestows an advantage over specialization.

## Introduction

In source-sink metapopulation scenarios, optimal habitat supports highly productive adults but the local demographics of sinks result in low contribution to the metapopulation usually because of marginally suitable habitat conditions [1]. Selection will favour the traits of the source population unless there is some reproductive contribution to the metapopulation from the sink [2], [3]. A fragmented landscape usually presents variable habitat patch quality for inhabitants in which the proportion of optimal to marginal habitat can be very low. This landscape may encompass enough environmental variability to invoke phenotypic response in individuals of a deme [4]. Thus, extensive marginal patches may constitute a relative sink with some genetic contribution especially when phenotypic adaptations enhance fitness in that sink. The extent to which that response is a genotypic adaptation will depend on factors such as relative habitat frequency and migration among habitats [5]. An alternative adaptation is phenotypic plasticity in which one genotype produces distinct phenotypes under different environmental conditions [6]. A plastic response to marginal conditions can increase individual fitness to support some genetic contribution to the metapopulation [7], [8]. In the context of developing conservation strategies, recognizing sources for critical species and the role of sinks in their maintenance is an important aspect [9], the source of the variability notwithstanding.

The habitat at hydrothermal vents is highly variable in space and time. Patches are controlled by subsurface fluid flows that can change abruptly with tectonic movements or volcanic activity [10], [11] that may affect an entire field. However, across a vent field, environmental dynamics tend to be independent as flow adjustments are localized. At these small scales, fluid flow in basalt cracks or through a sulphide chimney may shift causing death or invigoration of animal assemblages over the course of weeks [12], [13]. Vestimentiferan tubeworms (Polychaeta, Siboglinidae) often dominate faunal biomass at vents. They host bacterial symbionts in the trunk that depend on a sustained supply of dissolved sulphide delivered through the gills and vascular system from venting fluids. *Ridgeia piscesae* is the only tubeworm at vents on the ridges of the northeast Pacific (herein “Juan de Fuca Ridge”). This species dominates biomass and is a foundation species providing habitat for nearly all vent assemblages [14]. Gill filaments on the upper obturaculum emerge above the muscular vestimentum that wedges the animal in the tube. The trunk contains gonad and the trophosome organ housing the symbionts. Sperm masses are captured on female branchiae and fertilization in *R. piscesae* is internal; oocytes are released from the upper ovisacs and begin cleavage after release with ensuing pelagic larval development of several weeks [15]–[17].

Variation in *Ridgeia piscesae* is well-documented beginning with the initial description of two distinct species by Jones [18]. From morphology, Galkin [19] identified a continuous range of characters encompassing the original descriptions of *R. piscesae* and *R. phaeophiale*. Morphologies ranging from “short-fat”, “small-contorted” to “long-skinny” correspond to habitats of different temperatures [20]. When allozymes provided no genetic evidence basis for differentiation between these morphotypes, *Ridgeia* was re-described as a single species [21]. Subsequent targeted tests using a mitochondrial gene, a nuclear gene and fragment length polymorphisms could detect no genetic basis for the phenotypes [22]–[24]. Studies finding notable differences in levels of dissolved carbon and sulphide-binding amino acids between extreme morphologies invoked differential gene expression in different habitats [25], [26]; subsequently, the role of local environment in determining levels of haemoglobin gene expression was demonstrated by Carney et al. [27]. Liao et al [28] document metabolic “flexibility” in the use of nitrate by symbionts in conditions of differing ammonia levels in the fluid environment around different *R. piscesae* morphotypes and suggest both partners in the association are involved in optimizing the fitness of the symbiosis in different environments. Puetz [29] examined the same specimens that we study below (“Hi/Lo” pairs) to find strong support for a single gene pool at our main study site and no consistent differences among phenotypes in a mitochondrial gene region. Vrijenhoek [30] proposes that the morphological variability is a phenotypically plastic response to habitat. However, as transplant experiments to monitor growth change in different conditions are difficult because of handling damage, evidence for phenotypic plasticity rather than local adaptation remains circumstantial.

The objective of this work is to define how the variable hydrothermal fluid environment influences growth, body characteristics and reproductive condition in *Ridgeia piscesae*. We test the hypothesis that, in higher temperature habitats with greater dissolved sulphide flux, *R. piscesae* is fast-growing, short-lived and has a distinctive morphology with high reproductive fitness compared to animals in habitats of low fluid flux. We explore the components of fitness that may be enhanced by phenotypic variation in different fluid settings. We assess the reproductive condition of tubeworms in a variety of habitats to estimate the likely contribution of those habitats to the metapopulation. As metals markets push deep-sea prospecting into hydrothermal vents, designing reserve areas for the unique species inhabiting the deposits is a rising concern [31]. The means and extent to which a foundation species can function over a large habitat range is relevant to resilience in, and restoration of, degraded ecosystems.

## Materials and Methods

**Figure 1:**
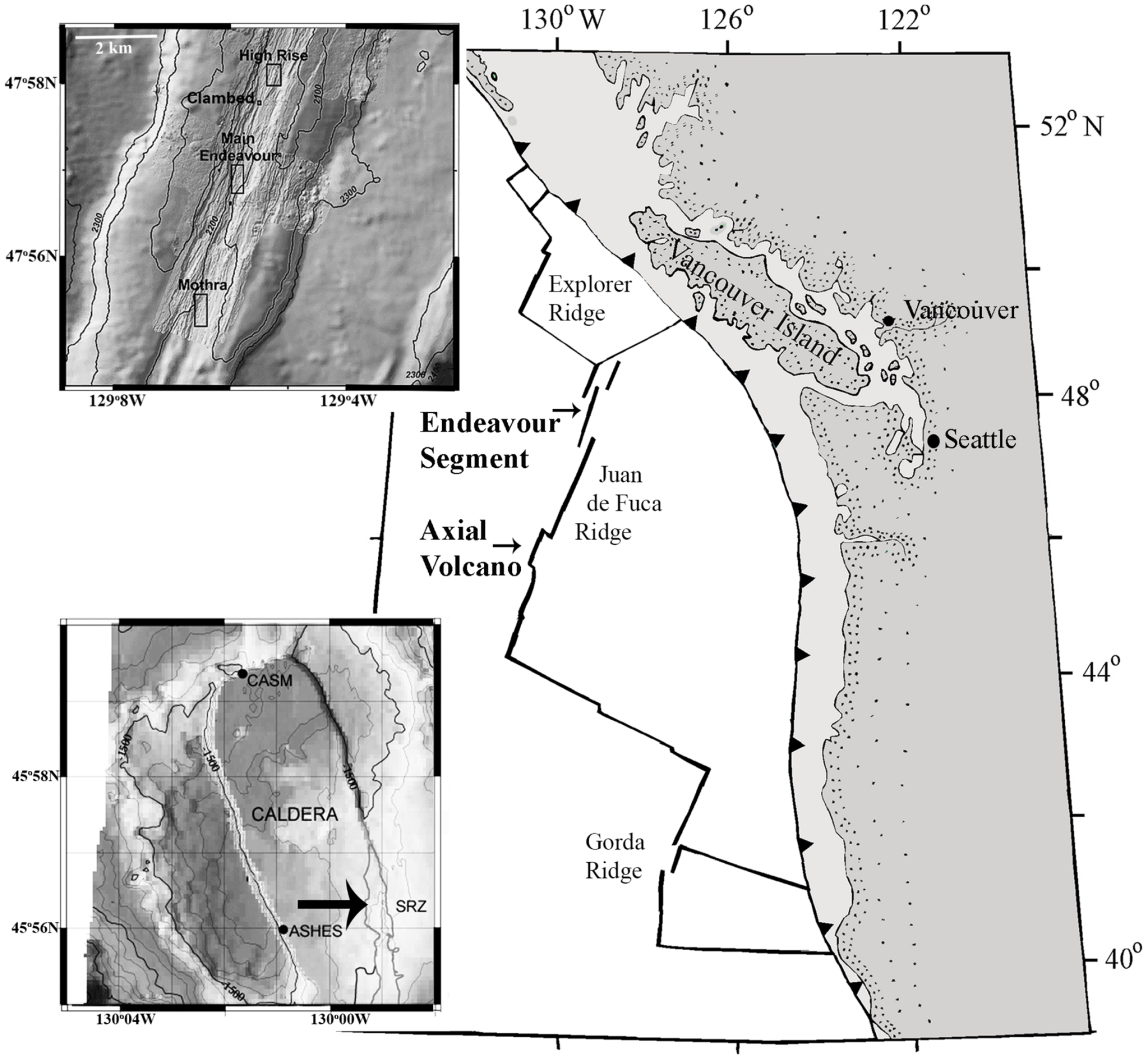
Locations of sampling sites on Juan de Fuca Ridge. Endeavour inset shows the three sampled vent fields: Clam Bed, Main Endeavour and Mothra. Axial inset shows locations of samples from three fields and the grey line near SRZ (arrow) delineates the extent of the 1998 lava flow.

The hydrothermal vents on Juan de Fuca Ridge (Figure 1) are located at discrete sites on separate segments of the ridge. Axial Volcano is one such site where vents within the caldera at ∼1550 m depth are short-lived due to lava eruptions. On Endeavour Segment, venting in the axial spreading valley is concentrated in several “vent fields” where fluids emerge through mineralized chimneys and through cracked seafloor basalts. Extrusive volcanism has not occurred within observation history here. Fields, separated by about 2 km, have multiple black smoker chimneys, clusters of animals around vent openings, and scattered tubeworms in low density. Collections came from Clam Bed, Main Field and Mothra Field at ∼2200 m depth (Figure 1). Deep-sea access was provided by the remotely operated vehicle *ROPOS* and the occupied vehicle *Alvin*, both of which used advanced force feed-back manipulators for collecting samples. Collection permits for this work were issued by Fisheries and Oceans Canada.

The terms “short-fat” and “long-skinny” are present in the literature for two morphotypes of *Ridgeia piscesae*, however, we also sampled other forms. To avoid these terms, we use “high flux” and “low flux” to indicate collection habitat following Sarrazin and Juniper [32] and Bates et al. [33] who define visual cues for emerging fluid of relatively high temperature and dissolved sulphide: shimmering, turbulent water. “Low flux” venting fluids are rarely visible but marked by clustered vent animals at low temperatures. Four sets of samples (totalling 731 individuals) were collected during seven sampling opportunities to study growth and condition in different venting settings and address these questions (see Table 1):

**Table 1:** Sample collection and preservation data for the four questions addressed.

Question	Year	Site	# samples	# worms	Preservation	Comments
**Tube growth post settlement**	1998	Axial	7	74	BF to 70% EtOH	Sampled 8 months post eruption.
	1999	Axial	2	52	BF to 70% EtOH	Resampled recruits at two new vents
	2000	Axial	2	80	BF to 70% EtOH	And again the following year
**Gonad and trophosome development**	1998–99	Endeavour	5	94	BF to 70% EtOH	Combined samples from chimney walls
**Condition in high and low flux**	2008	Endeavour	16	378	95% EtOH	8 pairs of “high flux” and “low flux” samples collected in close proximity
**Lipid content, gamete staging**	2009	Axial	3	35	−80°C, BF	Equal sex numbers
	2009	Endeavour	1	18	−80°C, BF	Equal sex numbers

BF = 10% buffered formalin; EtOH = ethanol.

1. How does initial *Ridgeia* growth respond to habitat conditions? On January 28 1998, an eruption on Axial Volcano extruded new lavas where a field of *R. piscesae* had existed the previous year [11]. Vents formed on the new lavas while some *R. piscesae* survived on the adjacent old lavas. In August 1998, we sampled newly settled worms at four new vents (Nascent, N41, M113, T&S), and survivors at two old vents (Oldwrms, Lrgwrms) nearby and one more distal (Bob) (Figure 1; Table S1). At each vent, fluid was assessed for maximum temperature and dissolved sulphide [34]. Markers were placed to pinpoint the site and in summers of 1999 and 2000, Nascent and N41 were re-sampled.

2. How do gonad and trophosome development relate to size? Five samples were collected in 1998 and 1999 from Main Endeavour Field and Clam Bed (Figure 1), preserved in 10% seawater formalin and transferred to 70% ethanol. Vestimentum width was used as a standard size measure following Thiébaut et al. [35]. Using a random stratified sampling design, the trunk of each individual was divided in 10 equal sections from which a 1 mm segment was chosen. The 10 segments were dehydrated in 90% propan-2-ol overnight followed by nine hours in 100% propan-2-ol with solution changed every 3 hours, then cleared with 100% xylene and impregnated in wax at 70°C. Subsequently, 5 µm sections were stained with Mayer's hematoxylin and eosin. One section from each segment was digitised using a binocular microscope to measure gonad and trophosome areas (Jandel Scientific's SigmaScan-Pro v4.01).

3. How do body and reproductive condition compare in different habitats? In June 2008, we systematically sampled vents in three vent fields of Endeavour Segment where a range of morphological types of *R. piscesae* lives in close proximity. We paired the samples, “Hi and Lo” (Table 1), such that collections in a pair were within 4 m and located on the same structure or seafloor crack; thus, the fluid source was the same but delivery rate differed. Temperatures were measured at the branchial plumes of *Ridgeia* when the probe found the highest consistent measurement for over a minute. Tubes were grasped by manipulator at the base and moved to closable boxes for recovery. The 25 largest worms were selected, the tubes split and preserved in 95% ethanol. In these Hi/Lo samples, the largest worms are most likely to be reproducing individuals that represent the high end of output from that location; this conservative approach reduced the differences between sample pairs as most worms in Lo samples were not reproductive. The following characters were assessed first: upper tube diameter, vestimentum diameter, obturaculum-vestimentum length, trunk length, wet weight of total body and of trunk alone, branchial plume condition and presence of sperm bundles in the vestimental groove. In some low flux specimens, the base of the trunk was missing so length is a conservative estimate. Plume condition was rated as: still developing, or little to extreme predation (0 to 3). Non-parametric tests (Mann-Whitney) in ‘R’ software examined differences between Hi and Lo characters. We generated the logistic curves in ‘R’ then optimized the logit model with the Excel Solver add-in. A Principal Components Analysis used normalized data with a maximum of five axes to determine common groupings.

The trunk was opened to determine gender and gonad development. An animal was deemed “non-reproducing” if gametes were absent in the anterior ovisacs or sperm sacs (but gametes might be seen in the gonad). To assess the relevance of blotted wet weight as a measure of trunk condition, 36 worms were processed for ash-free dry weight by first drying then burning; ash-free dry weight was highly correlated to dry weight (r^2^ = 0.996).

4. Do lipid content and gamete development reflect habitat? In summer 2009, additional specimens were collected from four sites at Axial and Endeavour (Table 1). The largest 4 males and 4 females from each site were frozen at −80°C. Lipids (a measure of gonad investment; [36]) were extracted by homogenizing bodies in chloroform and methanol then diluting to 2∶2∶1.8 (c:m:water) [37]. The lipid-containing layer was washed several times with a stock inorganic layer solution and dried under nitrogen at 50°C. Three formalin fixed females from each of high, moderate and low flux sites were sectioned at 0%, 30% and 60% of trunk length. We tested whether oocyte size varied among worms by measuring cross sectional area (Image J^©^ software) of oocytes sectioned through the nucleus within the ascending oviduct. Total gonoduct length was measured in the dissected females with a “packing distance” of 60 µm assigned to each egg along the gonoduct. The section counts were applied in three portions to derive an overall estimate of egg number as a measure of fecundity.

## Results

### *Ridgeia* growth at post-eruption vents on Axial

**Figure 2:**
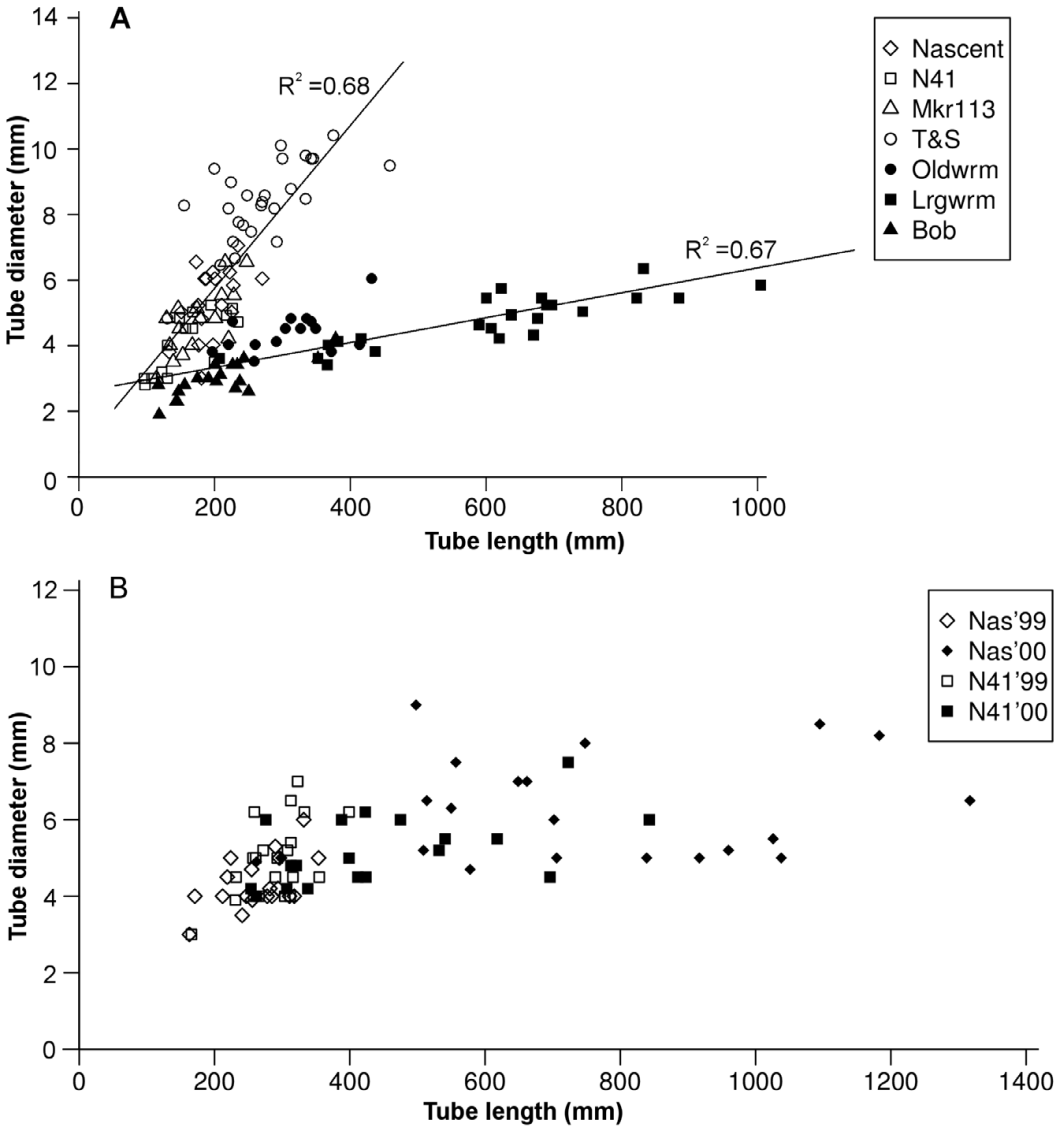
Tube characteristics of *Ridgeia piscesae* from Axial Volcano 1998 to 2000. The eruption in late January 1998 formed new vents rapidly colonized by tubeworm larvae. Growth in high flux is rapid and tube form is short and fat. As measured sulphide levels diminish through year 2000, tube form changes to resemble that of surviving worms on old lavas. A. Comparing *Ridgeia* samples from 8-month old vents on new lavas (open symbols) to *Ridgeia* from established vents (filled symbols). Lines are linear regressions on the new and old samples with Pearson correlation values shown. B. Largest worms at Nascent and N41 sampled at 18 and 30 months post eruption for comparison with 1998. In 1999, the tubes remain stocky; in 2000, a greatly elongated growth occurs in diminishing sulphide with little further increase in diameter.

**Figure 3:**
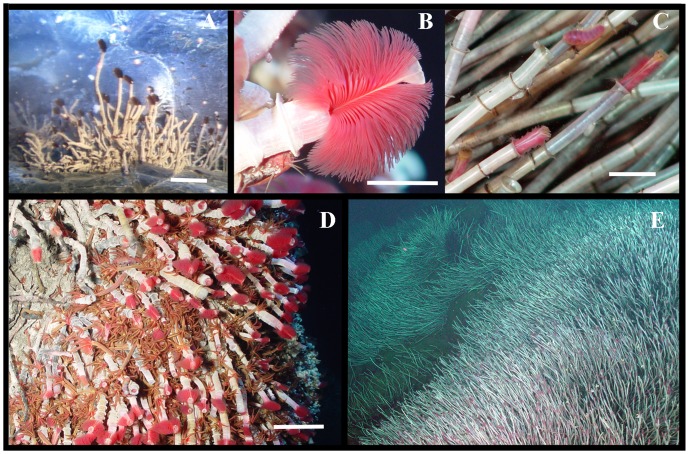
*Ridgeia piscesae* on Juan de Fuca Ridge. Images taken using the vehicle *ROPOS* (Canadian Scientific Submersible Facility).A. Eight months post-eruption at Nascent Vent, South Rift Zone, Axial; scale 10 cm. B. Branchial plume with white obturaculum of high flux *R. piscesae*; scale 1.5 cm. C. Sparse branchial plumes in low flux grazed by polynoid polychaetes (top centre); scale 1.5 cm. D. Clump of short-fat *R. piscesae* on the side of a smoker chimney; fluids emerging from ledge below. Orange polychaetes are *Paralvinella palmiformis*, a microbial grazer; scale 5 cm. E. Extensive tubeworm clumps on basalts in weak fluid flow between chimneys; image about 2 m across.

New vents created by the eruption were emitting fluids over 20°C with substantial levels of dissolved sulphide (Table S1) while surviving vents were cooler. In general, there is more sulphide at higher temperatures, a relationship evident across a much wider suite of samples at Axial [34]. In the following two years, heat and sulphide levels dropped as the volcano cooled. In the first year, tubeworms at these new vents were fatter and shorter than the older animals in less vigorous flow (Figure 2A). Settling larvae recruited and grew to 23 cm length within 280 days (Figure 3A) at which time, of 59 animals, 60% were already producing gametes. In the following two years as sulphide flux diminished somewhat, growth remained vigorous (at a minimum of 50 cm/yr) but the tube form elongate greatly (Figure 2B). As tubes lengthened, so did the trunk (r^2^ = 0.96, n = 57). One collection at Nascent Vent in 2001 revealed that all long worms had died and empty tube surfaces hosted a second generation of recruits over 30 cm long. The first generation lasted three years.

### Maturation of Ridgeia piscesae

**Figure 4:**
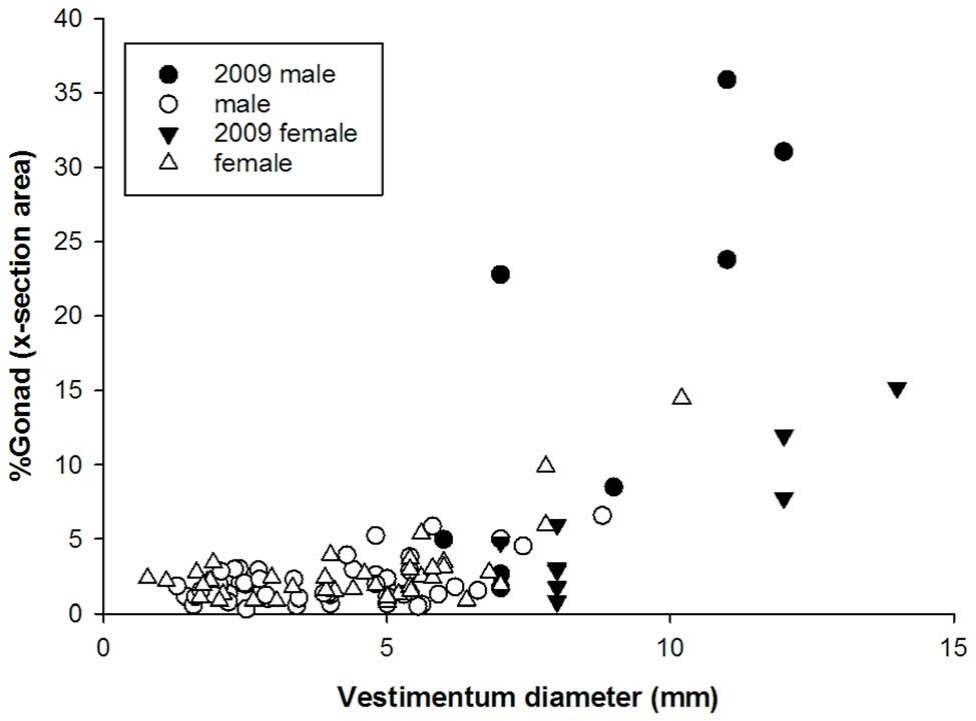
Estimate of gonad extent with increasing size. The majority of animals in our initial samples were small with little gonad development in both sexes (open symbols). Each measure of gonad area is the mean of 10 cross-sections equidistant along the trunk. Several worms from the 2009 study (filled symbols) augment numbers of larger individuals to illustrate gonad extent in full maturity.

We examined trophosome, gonad extent and gamete formation (Table 1) with onset of reproductive maturity in smaller animals. In 19 of 43 females and in 16 of 51 males, no gametes were seen in the sections; not until over 4 mm diameter did a majority of the females have oocytes when gonad began to enlarge. Only four individuals were over 7.5 mm diameter in this sample set but comparison with *R. piscesae* from the 2009 collection shows at larger sizes (over 10 mm vestimentum diameter) there is a marked increase in gonad extent, most notable in males (Figure 4). Trophosome area increased variably with vestimentum size (Spearman r = 0.58, p<0.01) to a maximum of 50% of the body area; there was no significant correlation with gonad size.

### Morphological variation between high flux and low flux habitats

Short, fat *R. piscesae* in vigorous flows at Endeavour occurred mostly as small clumps on sulphide chimneys with black smokers nearby (Video S1). Temperatures for these samples ranged up to 30°C (Table 2); in 1995, we had scanned the site of the HiC sample to measure sulphide levels around 90 µM sulphide at 18°C. Tubes were white and parchment-like with bright red branchial plumes that extended from nearly all individuals (Figure 3B, D). In contrast, small worms in lower temperatures with little visible flow were much more abundant on these chimneys while the longest worms grew on basalt, forming extensive “fields” (Figure 3E). Here, the obturactula had pale branchiae with very short lamellar filaments (Figure 3C) and were capped with chitin-like plates.

**Table 2:** *Ridgeia* sample location and body data for 2008 paired samples from Endeavour Segment.

Site	Sample	Temp	N	Vest Width	Sig	Trunk Weight		Trunk/OV Weight	Sig	Sperm Bundles	Repro-ductive
		°C		mm		gm		ratio		♀, ♂	%
**Clam Bed**	HiA	27.0	25	8.1 (0.2)		3.29 (0.31)	**	2.42 (0.17)		7, 3	96
	LoA	2.4	18	5.5 (0.2)		1.30 (0.17)		2.56 (0.23)		2, 0	67
	HiB	n/a	25	4.8 (0.1)		0.47 (0.05)	**	1.35 (0.07)	**	2, 0	44
	LoB	n/a	20	4.5 (0.1)		0.76 (0.08)		2.34 (0.18)		1, 0	30
**Main Field**	HiC	10.0	25	8.0 (1.0)	**	2.15 (0.16)	**	2.17 (0.13)	**	8, 9	96
	LoC	5.0	23	4.3 (0.6)		0.05 (0.01)		0.88 (0.06)		0, 0	0
	HiD	30.0	25	5.4 (0.2)	**	0.55 (0.05)	**	1.37 (0.08)	**	2, 0	92
	LoD	3.6	24	2.5 (0.1)		0.03 (.003)		0.69 (0.07)		0, 0	0
	HiE	30.0	20	7.6 (0.2)	**	2.02 (0.17)	**	2.00 (0.15)	**	4, 2	100
	LoE	11.4	26	4.3 (0.1)		0.34 (0.03)		1.31 (0.11)		2, 0	23
	HiF	n/a	25	7.8 (0.2)	**	2.52 (0.05)	**	2.43 (0.16)	**	9, 11	100
	LoF	n/a	25	4.3 (0.1)		0.38 (0.05)		1.32 (0.09)		0, 0	12
	HiG	n/a	24	7.4 (0.2)	**	1.79 (0.19)	**	2.02 (0.17)		4, 3	100
	LoG	n/a	24	4.9 (0.1)		0.87 (0.05)		2.07 (0.11)		0, 0	20
**Mothra**	HiH	21.3	25	6.0 (0.3)	**	0.90 (0.12)	**	1.68 (0.09)		5, 0	100
	LoH	5	25	3.4 (0.1)		0.14 (0.02)		1.25 (0.13)		0, 0	0

Temperature is the highest measured near the branchiae of the sampled individuals (n/a: probe not available). Body measurements are mean (st. err.) values for the sample. Vest  =  vestimentum; OV  =  obturaculum + vestimentum. ** sig p<0.01 Wilcoxon rank (vertical asterisks: Lo value is greater than Hi). The number of sperm bundles within the vestimentum fold is shown for females and males. An animal is considered reproductive if there were gametes in the anterior ovisacs or sperm sacs. The largest animals in each collection were used to maximize reproductive condition.

n/a  =  not available.

** sig p<0.01 Wilcoxon rank that Hi-Lo pairs differ; * p<0.05.

**Figure 5:**
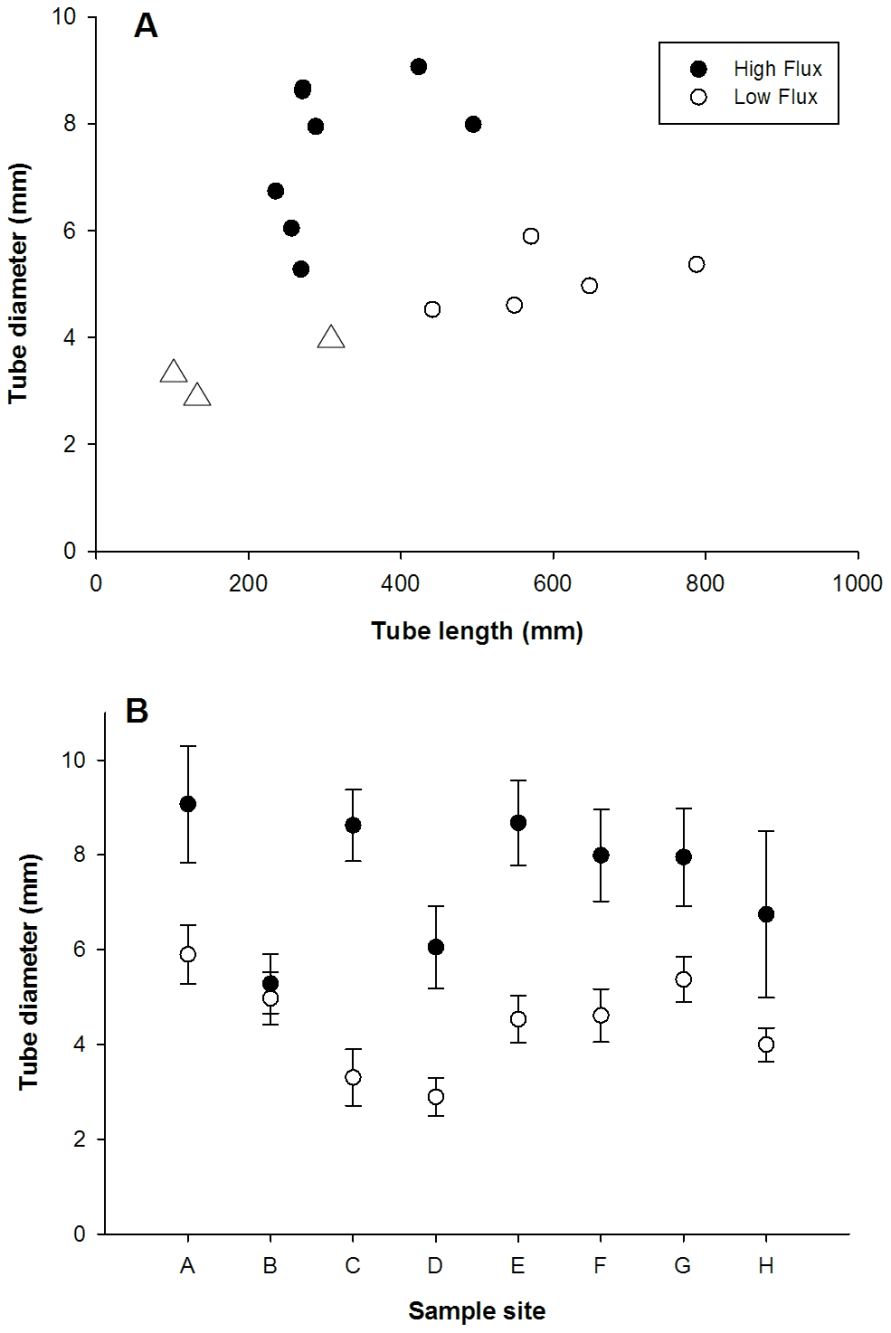
Comparison of tube characteristics between high fluid flow and low flow samples. Each sample represents 20 to 25 of the largest tubeworms to capture maximum growth extent. Tube diameter is highly correlated with vestimentum diameter and usually about 400 µm larger. A. Sample means plotted against tube length for comparison with Figure 2 illustrate that low flux animals are smaller diameter at length. Triangles are low flux samples in which all individuals were non-reproductive and deemed immature: LoC, LoD and LoH. B. In paired samples at each site members of each pair are within metres on the same structure. Bars are standard error. All Mann-Whitney paired tests show significant difference (p<0.01) between high and low flux samples except Site B.

Tubes from the Endeavour collections (total N = 379) showed width/length characteristics that were distinctive between high and low flux with the former resembling the Axial post-eruption worms (Figure 5A). Specimens from high flux samples were significantly wider and shorter than low flux in all but one sample (Figure 5B). The muscular vestimentum constructs the tube thus there is a strong correlation between anterior tube diameter and vestimentum width for both high flux (r^2^ = 0.91) and low flux (r^2^ = 0.96) tubeworms. Vestimentum width in high flux individuals was significantly larger than low flux (p<0.05, Mann-Whitney) for all pairs except one site. Similarly, most characters measured between pairs differed significantly with site G showing smaller differences (Table 2; Table S2). The longest worm was 60.5 cm in a 125 cm tube from LoA weighing 1.5 gm wet wgt; the heaviest worm was 6.0 gm with a body length of 19.6 cm in a 47 cm tube from HiG. Overall, the measurements defined wider, heavier but shorter worms from the high flux collections. Wet weight was a good predictor of trunk dry weight (dry = 0.142*wet +0.022; Pearson r^2^ = 0.91, n = 36).

**Figure 6:**
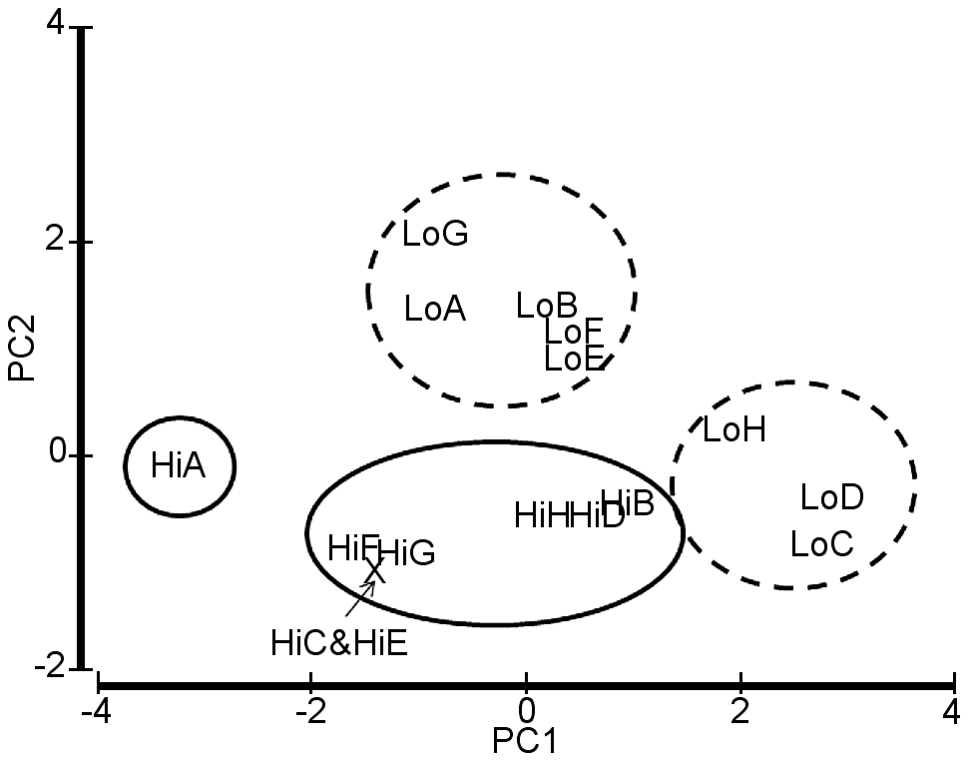
PCA plots paired samples based on morphological features. Using five principal components and four variables representing obturaculum-vestimentum and trunk size four groups of samples separate. PC1 explains 69% of the variability which increases to 96% with the addition of PC2. LoC, LoD and LoH were identified as immature juveniles; HiB also has many immature individuals.

Not all samples from high flux habitats were similar. In general, sample HiA had the largest worms measured by all characters while HiB, HiD and HiH were significantly smaller than other high flux samples. For most characters, the smallest individuals were from LoC, LoD and LoH (Table 2; Table S2). The remaining low flux samples were characterized by long, very thin trunks that left most of the tube space empty. A PCA using four characters (obturaculum-vestimentum length, vestimentum width, trunk length and trunk wet weight) identified four groups: Hi samples, Lo samples, HiA and the Lo samples that were immature (Figure 6). The first two axes accounted for 96% of the variability. The HiB, HiD and HiH trio, in which less than half the animals were reproductive, fall near the immature Lo group.

### Predation and Branchial Condition

Overall, 44% percent of the 379 worms had excellent branchial condition with well-developed lamellar filaments on the obturaculum and no evidence of predation. Within high flux samples, nearly all animals had the highest condition score (Table S2) reflecting the bushy red plumes in Figure 3 (B, D). The exception was HiB in which many animals had grazed lamellae. In comparison, animals from the low flux sites had evidence of predation including lamellae completely shaved on parts or all of the obturaculum and chunks of tissue missing. For worms in samples LoC, LoD and LoH, and also in half of the LoB sample, we determined that nearly all branchiae had the distinctive features of a developing juvenile [18]. Most Lo worms had many chitinous plates on the obturaculum apex to plug the tube as an anti-predator device; these plates were rare in Hi worms. In one collection pair, we assessed the presence of predators: the HiC and LoC collections yielded 562 and 720 other macrofauna, respectively. Polynoid polychaetes, mostly *Lepidonotopodium piscesae*, were the predators: in HiC they constituted 4% of the wet weight of this additional fauna while in LoC, they were 22% of the wet weight (26 individuals) thus reflecting field observations of higher abundance in lower flux conditions.

### Reproductive Features and Body Condition

We observed tethered sperm bundles streamed from worms in high fluid flux several times. Ovoid bundles attached to strands up to 15 cm long bounced vigorously in the turbulence over the branchial plumes (Video S1), each traceable to an individual worm. Bundles showed no swimming or mobile behaviour, thus fertilization appears to occur between individuals within strand range. Among the sampled worms, 37% of high flux females and 36% of high flux males held sperm bundles compared with only 5% of low flux females and 0% of low flux males. All high flux samples had at least some females with sperm masses in the vestimental fold while only three low flux sites had females with masses (Table 2). In most high flux tubeworms, the gonad was full of gametes and occupied a large part of the trunk whereas in low flux animals most had little gonad and, in some low flux tubeworms, only the top of the gonad held gametes. All males and over 80% of the females from high flux sites had gametes in the upper storage sacs and were deemed to be reproductively active with the exception of sample HiB from Clam Bed (Table 2). From low flux sites, no worms were reproductive in samples LoC, LoD or LoH (about 20% had some gametes). High flux samples had a significantly higher proportion of reproductive individuals than low flux samples (Mann-Whitney test, p<0.05).

**Figure 7:**
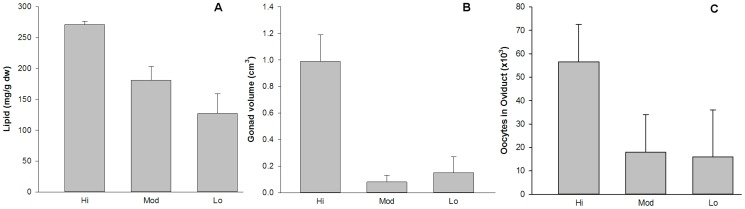
Female reproductive condition in *Ridgeia piscesae* in high, moderate and low flux habitats. Indicators reflect that females from the high flux samples have greater reproductive output; bars are sd. A. Total lipid content in trunks of 12 individuals.B. Gonad volume estimated from trunk sections. C. Total oocytes estimated from section counts and gonad volume. Neither moderate nor low flux worms had oocytes in ovisacs ready for release.

The largest lipid values occurred in females from a high temperature setting (26°C) (Figure 7A). Overall gonad volume was much larger in high flux individuals than moderate (10°C) or low (4°C) flux (Mann-Whitney, p<0.01) and oocytes in the oviducts more abundant in high flux (Figure 7B, C). The overall estimate of fecundity was 56,000 oocytes in high flux versus 27,000 in moderate or low when oocytes were present; however, only high flux worms had oocytes in the ovisacs ready for release. There were no significant differences among oocyte sizes from the three sites. Similarly, in males, the high flux animals displayed much greater amounts of gonad and trophosome as illustrated in the trunk cross-sections in Figure S1.

**Figure 8:**
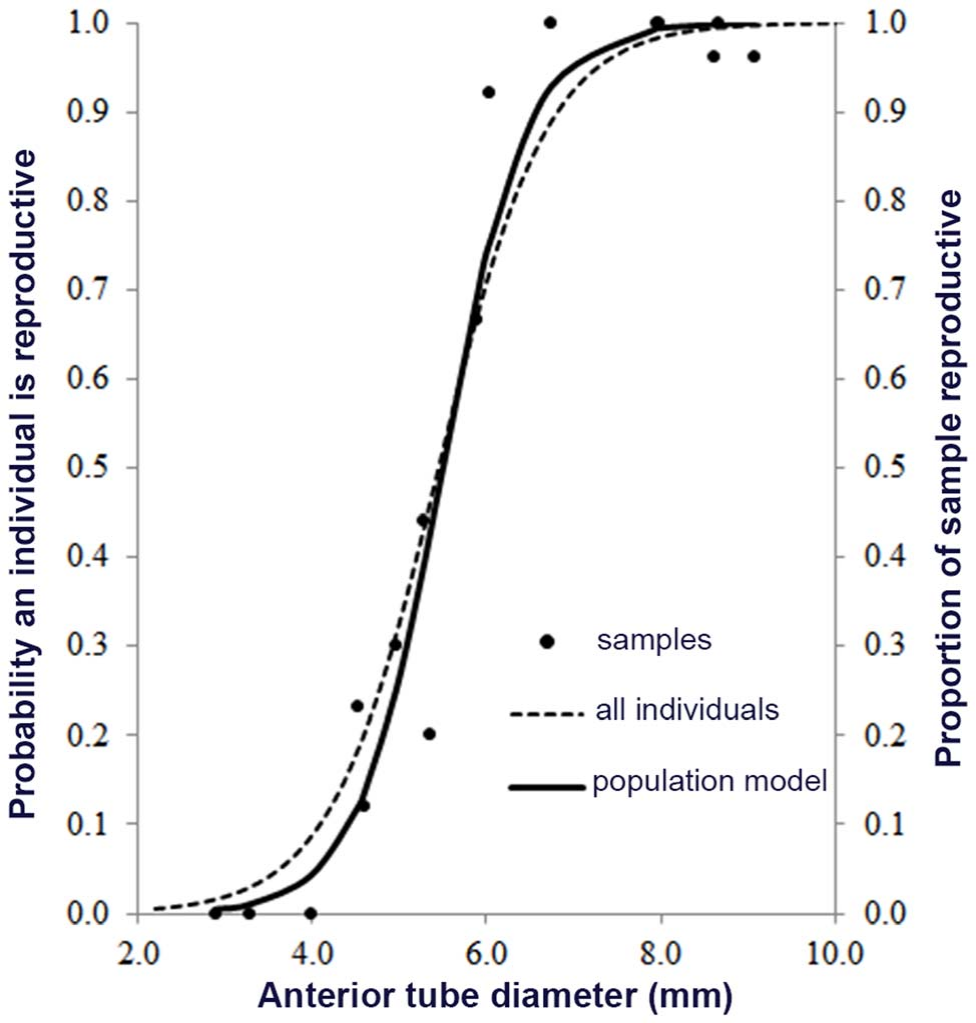
Model of reproductive readiness based on tube diameter. Onset of reproduction with size follows a logistic curve. The likelihood that an individual is reproductive at a given tube diameter is shown in the dotted line (left axis) using all individuals in our 2008 study. Tube diameter is highly correlated body characters but is an easier trait to measure. The right axis represents the proportion of the Endeavour samples that were reproductive (dots) and the full logistic curve (solid line) is the best fit for any sample taken.

Of the 379 worms assessed for developmental state in the paired samples, 171 were considered non-reproducing with empty ovisacs or seminal vesicles; all these animals, except two, were under 6.7 mm tube diameter. All other tubeworms over 6 mm diameter were reproductive with abundant gametes. Figure 8 illustrates that an individual is likely to become reproductive between 5 and 6.5 mm diameter based on a logistic model that fits the observations well (Hosmer-Lemeshow test of goodness of fit, p<0.01). The model combines all individuals, however, the likelihood of individuals being reproductive between the size of 5 and 6.5 mm was significantly lower in low flux samples (Chi-Sq; p<0.01). The proportion of reproductive individuals within a sample follows the logistic curve, p = 1/(1+EXP(−1*(−11.3+2.1*ATD))) where ATD is anterior tube diameter (Figure 8).

## Discussion

A major feature of the *Ridgeia piscesae* population is morphological variation that corresponds to habitat: fast-growing, large animals have high reproductive success and fecundity in higher temperatures ( = dissolved sulphide, as in Butterfield et al. [34] and Urcuyo et al. [38]). Long, thin worms with low fertilization and reproductive readiness occur in low temperatures. Abundant small intermediate forms are mostly immature. Morphological differences relate to relative fitness in different habitats. While the primary environmental driver of variation is the flux of dissolved sulphide, a secondary driver is habitat stability as locations (or times) of high flux may be short-lived or subject to substratum disruption. Recruitment, growth and maturation of a short-fat tubeworm phenotype happens within months after volcanic eruption when temperatures were relatively high. On Axial, and also at an adjacent eruption site in 1994 [39], *R. piscesae* grew at rates over 50 cm per year and were dead after three years. The tube character changed as sulphide decreased, becoming long and recumbant, likely to bring the branchiae closer to the emerging fluid. On sulphide chimneys, fluid redirection opens new vigorous outlets inducing colonization and high growth rates or causing abrupt death due to high heat or substratum instability [20]. Where low flow is sustained for long periods, tubeworms can grow over 2 m in length as at Clam Bed. Here, growth of marked individuals is very slow; using the upper 10% of measured rates, Urcuyo et al. [40] give conservative estimates of life-spans between 10 and 40 years stating larger animals are likely older.

The branchial plume of the vestimentiferan is the uptake site for oxygen and metabolites that support the symbiotic bacteria in tubeworms [25]. Predation, however, takes a large toll on low flux *R. piscesae* plumes; Urcuyo et al. [38] note predation on *R. piscesae* from Clam Bed at ∼95% frequency. They also determine that this phenotype can take up sulphide through a thin-walled posterior extension of the tube that accesses this critical nutrient where hydrothermal fluid exits basalt cracks. In large low flux worms, we see a long trunk that would increase potential uptake area especially when branchiae are reduced. This functional adaptation that reduces reliance on the branchiae is facilitiated by backward growth of the tube and trunk.

Our novel observation of the sperm mass trailing from males in turbulent flows completes our understanding of the fertilization process begun by Southward and Coates [15] and MacDonald et al. [17] and underscores the importance of branchial capture. We find no evidence of periodicity in gametogensis so assume that fertilization occurs when sperm are available. The presence of oocytes in ovisacs indicates reproductive readiness in females, however, not all released oocytes develop to embryos, especially from small females [16]. Many traits of fitness indicate that the high flux, short-fat phenotype has a high contribution to the next generation compared to other phenotypes (long, thin or short) (Table 3). “Live long and prosper” is not an option for this species in a time-and-space variable environment. Three scenarios appear to exist: i) early maturation, reproduction and death in high nutrient habitat, ii) slow growth and long life with low reproductive output sustained by regular low sulphide supply, or iii) remaining a small form that never reaches maturity due to inadequate nutrient flux, predation and/or habitat instability. While it is possible that, over time, oocyte output from a long-lived tubeworm could approach that of a short-fat phenotype, our data suggest that fertilization success is likely very low in worms with predator-cropped branchiae. Thus, low flux habitat is occupied by worms with minimal reproductive output while the high flux habitat patches support high generational turnover with constant contribution of progeny.

**Table 3:** Traits in *Ridgeia piscesae* that influence individual fitness.

Functional Trait	Attribute	Fitness Component	Notes
Threshold reproductive size	smaller in Hi	Reproductive probability	diameter significantly different between phenotypes
Maturation rate	sooner in Hi	Reproductive probability	months versus years between phenotypes
Growth rate	faster in Hi	Reproductive probability	varies over two orders of magnitude among phenotypes
Gametic tissue allocation	greater in Hi	Embryo production	increases with size; high flux phenotype grows larger
Physiological condition	variable	Embryo production; Survivorship	lipids greater in high flux but see adaptation of haemoglobin levels and trunk dimensions in low flux phenotype
Branchial condition	better in Hi	Embryo production; Survivorship	healthy branchiae enhance metabolite uptake and fertilization; growth of defense structures in low flux phenotype
Aging rate	slower in Lo	Survivorship; Recruitment	low turnover (decades) in low flux enhances individual fitness and provides long-term settlement surface for recruits.

The main environmental driver is the level of dissolved sulphide flux to sustain the bacterial symbionts. Habitat stability, which is low in high flux habitat, is likely an additional factor. This study did not assess factors that influence recruitment success (a fitness component). Most attributes are assessed in this study but additional literature information includes growth rates [38], [39], hemoglobin levels [27] and sulphide uptake [38].

The demographic potential of a patch in a metapopulation depends on the quality of the underlying habitat [41]. The sulphide-rich habitat of robust, reproductive *Ridgeia piscesae* is both limited and unstable. It occurs mostly in small patches on black smoker chimneys where new heat bursts and smoker growth sponsor rare, short-lived clumps of worms or in bursts of heat/sulphide through basalt after tectonic or volcanic events. On large chimneys, small tubeworms occupy much of the surface. Sarrazin and Juniper [32] mapped the structure that we sampled a decade later (Vent “C”); low temperature *R. piscesae* dominated biomass but 95% of those they measured were under 7.5 cm body length, thus very small diameter. Only our LoD worms were this small – animals that were non-reproductive. Even in trying to find large low flux *R. piscesae*, we still collected immature samples from chimneys. The evidence that the *Ridgeia piscesae* metapopulation at Endeavour has a source-sink structure is strong. The source is optimal high sulphide habitat that is limited but has a high throughput of very productive animals, thus the rate of offspring production per m^2^ is far higher than low flux habitat. Habitat surveys on seven chimneys document about 2% as high flux tubeworm habitat compared to over 80% of the surface with low flux worms [42]. Thus, while the sheer abundance of low flux worms on chimneys is substantial, the animals rarely mature before flow ceases; repeated mapping illustrates these surface changes [12], [42].

On basalts, we estimate 2% of the tubeworm coverage as high flux phenotypes from sporadic overflight imagery. Suitable high flux habitat is rare although Clam Bed is an interesting exception where the longest low flow worms also occur in a stable vent setting. Here, we find the highest reproductive condition for low flow among the selected large worms although predation was high and likely to impede fertilization. Urcuyo et al. [38] find over 70% of 500 worms from their Clam Bed grab were under 4.0 mm diameter and, therefore, not reproducing (Figure 8); the largest was 6.5 mm tube diameter, thus, overall reproductive output is limited to few individuals. Suitable high flux habitat is rare enough that contribution from low flow, long-lived animals is probable despite the finding that most low flux habitat animals are non-reproductive. Overall metapopulation fitness may be higher when most recruits are generated regularly from high fecundity, short-lived individuals in small habitat patches, but long-lived individuals in poor quality, stable habitat will store genetic diversity generated from successful recruits over many years. Indeed, the marginal habitats may perform a rescue function through a few offspring repopulating high flux habitat after times of high regional habitat instability.

Despite the high proportion of marginal habitat, we do not see fixation of the low flux phenotype as might be predicted if the phenotypes are a result of genetic specialization (e.g. Dias [43]). Given this point, the observations presented here, plus past studies, it is probable that variation is a plastic response to habitat. Phenotypic plasticity is adaptive when the appropriate phenotype in each environment has a higher fitness than the alternative phenotype [44]. If plasticity exists in *Ridgeia piscesae*, the morphological response is expressed in at least two ways: i) alteration of the trunk and tube to optimize the main site of sulphide uptake (branchiae versus tube base); and ii) formation of stiff caps on the obturaculum that plug the tube when predators are more abundant: an inducible defense trait. Additional physiological plasticity in levels of haemoglobin gene expression [27] and of certain amino acids [26] would relate to the mobilization of dissolved sulphide within the body. The plasticity cost to the low flux habitat worms lies in the resources needed to construct additional structural parts but the long, thin morphology confers greater fitness in low sulphide supply. An overall fitness is likely balanced by relative habitat frequency and the rate at which propagules are delivered to the gene pool. Plasticity is common in sessile organisms occupying a variable environment in which individual fitness can increase by modification of a morphological or physiological trait [45]. Given the nature of unpredicitability of high quality habitat, it is adaptive to maintain a reaction norm that includes fitness in low quality habitat so that part of the population will survive a major disturbance in this volatile setting. However, further work through reciprocal transplants is necessary to prove that a tubeworm can alter its form under different fluid flow conditions.

Consequences of phenotypic variation (plastic or otherwise) on ecological processes can be profound [46]. *R. piscesae* is a foundation species that provides structured habitat for many species in which tube surface area, tube form and surface character shape the associated communities as does tube longevity. The architecture of the tubeworm bush is dictated by phenotype; bushes with small twisted worms of several recruit generations host the greatest number of species while the long, thin phenotype hosts the least [14]. Thus, the indirect effects of a range of morphotypes on the vent community are notable.

Consideration of metapopulation structure including the disposition of sources and sinks should be a factor in conservation [43], [47]. This work was conducted with a Marine Protected Area with zoned uses under Canadian jurisdiction. We recommend that a management strategy include monitoring of human impacts on high flow habitat in all zones. As mining scenarios approach reality for the sulphide chimneys that form the habitat for many hydrothermal vent animals [31], application of appropriate strategies for biological reserves that support diversity protection and restoration potential is critical. Dynamic, changeable landscapes present further challenges for metapopulations in which suitable habitat persistence may be highly variable [48]. The cumulative effects of anthropogenic and natural disturbance bears further consideration in vent ecosystems.

## Supporting Information

Table S1 **Maximum temperature and dissolved sulphide concentrations from vents on Axial Volcano before and after the eruption of January 1998.** Measurements were taken in July to September of each year. *Vents with asterisk remained from pre-eruption; Sonne Vent was paved over by the eruptive lavas. n/a  =  not available.
(DOCX)

Table S2 **Additional measurements on** ***Ridgeia piscesae*** **from Endeavour Hi/Lo samples.** Body measurements are mean (st. err.) values for the sample. Obt-Vest  =  Obturaculum + Vestimentum. Predation is a qualitative scale from 0 (none) to 3 (major damage).
(DOCX)

Video S1 **Tubeworm sperm bundles released on long filaments in turbulent flow.** A large cluster of short-fat *Ridgeia piscesae* on a sulphide chimney at Endeavour Segment, Juan de Fuca Ridge (2190 m depth). The small black spire to the left is emitting hydrothermal fluid over 350°C. Initial image is about 1.5 m across. Zoom at 15 seconds shows tubeworms, alvinellid polychaetes and small white limpets. To the left, four sperm packets from *R. piscesae* are moving in the turbulent flow tethered to the males. Sperm packets are also present entangled in the red branchiae.
(MP4)

Figure S1 **Cross-sections through upper trunk of male** ***Ridgeia piscesae*** **comparing long-skinny to short-fat.** Scale bar is 1 mm. A. Long-skinny worm from Mod flux Site K (Axial). Trophosome is reduced and gonad is barely visible. (Vestimentum diameter, 7 mm.). B. Short-fat worm from high flux Site I (Endeavour). Trophosome and gonad occupy most of the trunk area. (Vestimentum diameter, 14 mm.). c – coelom; cf – coelomic fluid; dv – dorsal blood vessel; fm – feather muscle; g – gonad; t – trophosome.
(TIF)
