# Supplementary material for: Phenotypic Variation and Fitness in a Metapopulation of Tubeworms (Ridgeia piscesae Jones) at Hydrothermal Vents
Source: PLoS One. 2014 Oct 22;9(10):e110578. doi: 10.1371/journal.pone.0110578 (PMC4206443; doi:10.1371/journal.pone.0110578)
Supplement: Table S1 — Maximum temperature and dissolved sulphide concentrations from vents on Axial Volcano before and after the eruption of January 1998. Measurements were taken in July to September of each year. *Vents with asterisk remained from pre-eruption; Sonne Vent was paved over by the eruptive lavas. n/a = not available. (DOCX) [file pone.0110578.s001.docx]

**Table S1: Maximum temperature and dissolved sulphide from vents on Axial Volcano before and after the eruption of January 1998.**Measurements were taken in July to September of each year. *Vents with asterisk remained from pre-eruption; Sonne Vent was paved over by the eruptive lavas. n/a = not available.

| **Year** | **Vent** | **T_max_** | **H_2_S** |
| --- | --- | --- | --- |
|  |  | ^o^C | mmol/kg |
| 1997 | Sonne | 6.7 | 0.09 |
| 1998 | Nascent | 23.5 | 0.28 |
| 1998 | N41 | 22.7 | 0.08 |
| 1998 | M113 | 25.2 | 0.45 |
| 1998 | T&S | 20.3 | 0.18 |
| 1998 | *Lrgwrms | 16.1 | 0.11 |
| 1998 | *Oldwrms | 9.5 | 0.12 |
| 1998 | *Bob | 4.5 | 0.12 |
| 1999 | Nascent | 15.3 | 0.15 |
| 1999 | N41 | 15.3 | n/a |
| 2000 | Nascent | 14.7 | 0.06 |
| 2000 | N41 | 13.2 | 0.03 |
