# Supplementary material for: Phenotypic Variation and Fitness in a Metapopulation of Tubeworms (Ridgeia piscesae Jones) at Hydrothermal Vents
Source: PLoS One. 2014 Oct 22;9(10):e110578. doi: 10.1371/journal.pone.0110578 (PMC4206443; doi:10.1371/journal.pone.0110578)
Supplement: Table S2 — Additional measurements on Ridgeia piscesae from Endeavour Hi/Lo samples. Body measurements are mean (st. err.) values for the sample. Obt-Vest = Obturaculum + Vestimentum. Predation is a qualitative scale from 0 (none) to 3 (major damage). (DOCX) [file pone.0110578.s002.docx]

**Table S2. Additional measurements on *Ridgeia piscesae* from Endeavour Hi/Lo samples.**Body measurements are mean (st. err.) values for the sample. Obt-Vest = Obturaculum + Vestimentum. Predation is a qualitative scale from 0 (none) to 3 (major damage).

| **Site** | **Sample** | **Temp** | **N** | **Obt-Vest length** |  | **Trunk weight** |  | **Trunk/OV weight** |  | **Predation** |
| --- | --- | --- | --- | --- | --- | --- | --- | --- | --- | --- |
|  |  | ^o^C |  | mm |  | gm |  | gm |  |  |
| **Clam Bed** | HiA | 27 | 25 | 32.1 (1.8) | ** | 3.29 (0.31) | ** | 2.42 (0.17) |  | 0 |
|  | LoA | 2.4 | 18 | 23.7 (1.3) |  | 1.30 (0.17) |  | 2.56 (0.23) |  | 1.3 |
|  | HiB | n/a | 25 | 18.9 (0.8) |  | 0.47 (0.05) | ** | 1.35 (0.07) | ** | 1.4 |
|  | LoB | n/a | 20 | 19.8 (1.0) |  | 0.76 (0.08) |  | 2.34 (0.18) |  | 1.6 |
| **Main Field** | HiC | 10 | 25 | 22.7 (3.4) | ** | 2.15 (0.16) | ** | 2.17 (0.13) | ** | 0 |
|  | LoC | 5 | 23 | 13.0 (2.3) |  | 0.05 (0.01) |  | 0.88 (0.06) |  | n/d |
|  | HiD | 30 | 25 | 19.2 (0.9) | ** | 0.55 (0.05) | ** | 1.37 (0.08) | ** | 0 |
|  | LoD | 3.6 | 24 | 8.5 (0.4) |  | 0.03 (.003) |  | 0.69 (0.07) |  | n/d |
|  | HiE | 30 | 20 | 25.1 (0.9) | ** | 2.02 (0.17) | ** | 2.00 (0.15) | ** | 0 |
|  | LoE | 11.4 | 26 | 22.6 (1.0) |  | 0.34 (0.03) |  | 1.31 (0.11) |  | 1.8 |
|  | HiF | n/a | 25 | 23.3 (0.9) | ** | 2.52 (0.05) | ** | 2.43 (0.16) | ** | 0 |
|  | LoF | n/a | 25 | 21.5 (0.8) |  | 0.38 (0.05) |  | 1.32 (0.09) |  | 1.9 |
|  | HiG | n/a | 24 | 24.3 (1.0) | * | 1.79 (0.19) | ** | 2.02 (0.17) |  | 0.1 |
|  | LoG | n/a | 24 | 27.7 (1.0) |  | 0.87 (0.05) |  | 2.07 (0.11) |  | 1.3 |
| **Mothra** | HiH | 21.3 | 25 | 20.1 (1.1) | ** | 0.90 (0.12) | ** | 1.68 (0.09) |  | 0 |
|  | LoH | 5 | 25 | 12.6 (0.4) |  | 0.14 (0.02) |  | 1.25 (0.13) |  | n/d |
| n/a = not available; n/d = branchiae not developed so predation difficult to detect. | | | | | | | | |  |  |
| ** sig p<0.01 Wilcoxon rank (vertical asterisks: Lo value is greater than Hi); * p<0.05. | | | | | | | | | | c |
