# Supplementary figures and images for: Phenotypic Variation and Fitness in a Metapopulation of Tubeworms (Ridgeia piscesae Jones) at Hydrothermal Vents
Source: PLoS One. 2014 Oct 22;9(10):e110578. doi: 10.1371/journal.pone.0110578 (PMC4206443; doi:10.1371/journal.pone.0110578)

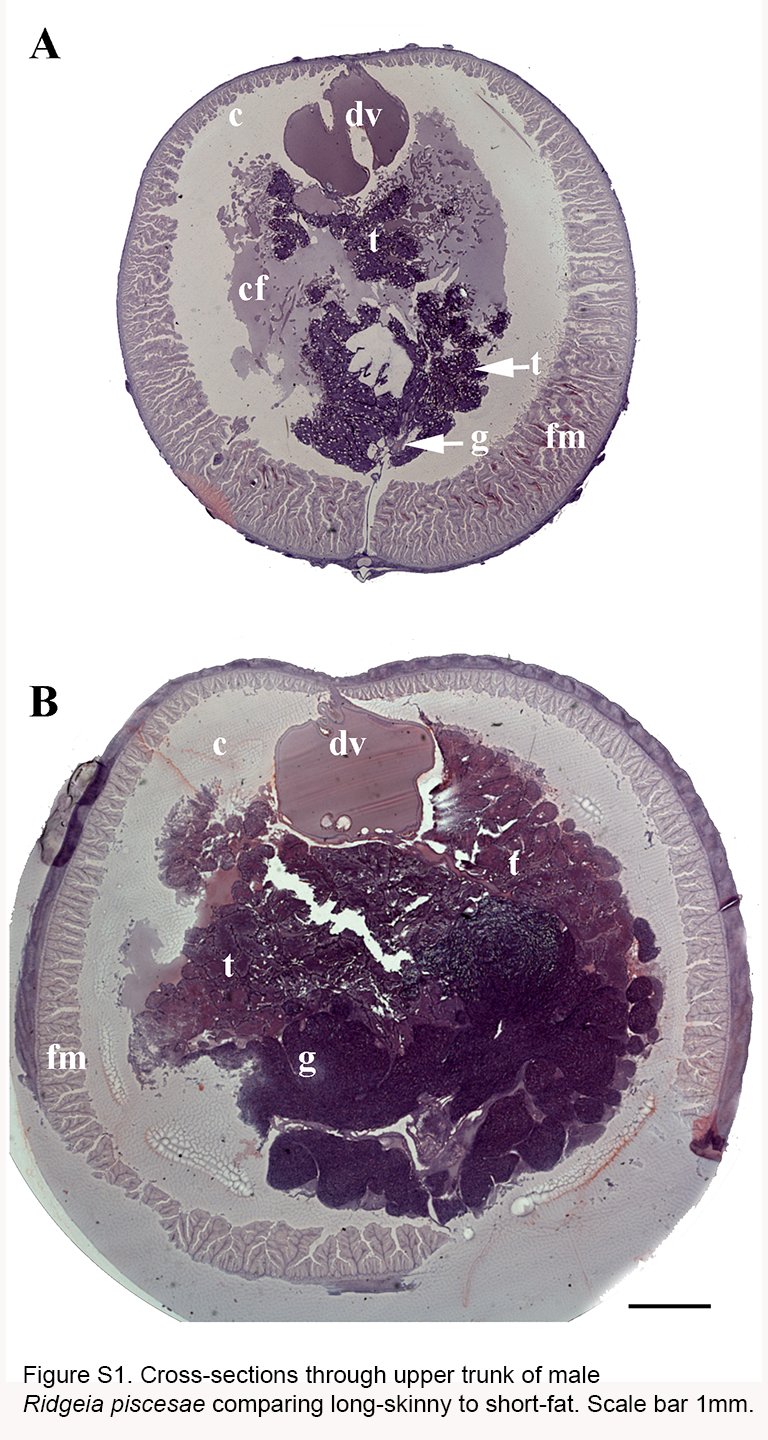

Supplement: Figure S1 — Cross-sections through upper trunk of male Ridgeia piscesae comparing long-skinny to short-fat. Scale bar is 1 mm. A. Long-skinny worm from Mod flux Site K (Axial). Trophosome is reduced and gonad is barely visible. (Vestimentum diameter, 7 mm.). B. Short-fat worm from high flux Site I (Endeavour). Trophosome and gonad occupy most of the trunk area. (Vestimentum diameter, 14 mm.). c – coelom; cf – coelomic fluid; dv – dorsal blood vessel; fm – feather muscle; g – gonad; t – trophosome. (TIF) [file pone.0110578.s004.tif]
